# Editorial: Emerging trends in innovation management and entrepreneurship development in the 21st century: issues, challenges, and opportunities

**DOI:** 10.3389/fpsyg.2023.1145727

**Published:** 2023-09-20

**Authors:** Minwir Al-Shammari, Waleed A. Aziz, Sajjad M. Jasimuddin

**Affiliations:** ^1^Department of Management and Marketing, College of Business Administration, University of Bahrain, Sakhir, Bahrain; ^2^Kedge Business School, Talence, France

**Keywords:** innovation, entrepreneurship, economic development, leadership, sustainability

## 1. Introduction

Constant improvements in digital technology have triggered a historic shift in how people live and work. Examples include the proliferation of digital ride-hailing platforms that rapidly grab market share from incumbent transportation providers and the emergence of entirely new industries, such as unmanned aerial vehicles based on digitized hardware that did not exist until recently. The rapidity and pervasiveness of this shift, as well as its worldwide influence, has prompted not just business owners and inventors but also government agencies throughout the world to evaluate and act on the consequences this shift may have on competition, value creation, and society at large (ACCC, 2019; Furman et al., 2019). In today's digital age, innovation and entrepreneurship refer to activities where digital technology is used for conventional business models. The revolutionary developments brought due to digital transformation are beneficial to both fields. Digital technologies, according to the literature (Jasimuddin et al., 2017; Nambisan, 2017; Al-Shammari and Waleed, 2018; Al-Shammari and Marhoon, 2022), dissolve traditional boundaries and shift the agency of entrepreneurship and innovation processes and outcomes, potentially rendering existing theories obsolete and necessitating investigation of these intersections as novel phenomena. The premise is that digital technologies are not merely another technological transition but fundamentally different from their analog predecessors (Gu and Wang, 2022; Xia et al., 2022). For instance, they can continually develop (Yusof et al., 2023) and overthrow established behaviors (Naeini et al., 2022; Pfotenhauer et al., 2022). As an innovative practice, Business Process Reengineering (BPR) continues beyond the implementation stage. Although most sources see BRR implementation as the last stage of BPR management, companies should keep it that way because modern businesses function in a volatile and ever-changing environment. Keeping the feedback loop open is crucial for the success of the reengineering effort. The established framework offers SMEs a paradigm for reengineering preparation and transformation into an ongoing activity rather than a one-off attempt (Aziz, 2019). The global impact of digital technologies on economic and social activities is something that not even visionaries like Jeff Bezos (the founder of Amazon), Sergey Brin and Larry Page (the founders of Google), or Steve Jobs (the co-founder of Apple) could have foreseen. Put another way; digital technologies may be viewed as external facilitators that stimulate and encourage processes or enable results in entrepreneurship and innovation (Von Briel et al., 2018). Products (Keränen et al., 2022), product or service platforms (Pattinson et al., 2023), infrastructure tools or systems (Aldrich, 2014; Nakshabandi and Jasimuddin, 2022), and digital applications, components, or media content are just a few examples of the functions and manifestations digital technologies may take on (Berger et al., 2021). This Research Topic expands the current understanding by bringing together entrepreneurship and innovation management experts. We begin by surveying what is known about entrepreneurship development and innovation management research; then, we explain how each piece adds to the body of knowledge and business practices. Then we suggest where the field may go from here in terms of new avenues for study.

## 2. Innovation management and organizational objectives

“Innovation management” refers to practices standardizing how innovative ideas, processes, and goods are developed and introduced into the market (Jasimuddin, 2012). Furthermore, small, medium and big businesses' performance benefits from this process (Melendez et al., 2019). When a firm aims to gain a competitive edge via innovation development, the innovation management process must be effectively implemented by formulating strategies and establishing an adequate administrative framework to back up the innovation (Melendez et al., 2019). In recent years, there has been a slew of qualitative research on innovation management. According to Morente and Ferràs (2017), a conceptual framework for innovation management was developed, focusing on the role of brokers in the process. To evaluate the effectiveness of innovation management in construction firms, Serpell and Alvarez detailed a mixed quantitative and qualitative strategy for doing so (Adeel et al., 2023). The concepts of effective innovation behavior and management in related organizations were uncovered in a different study by Frank (2019). Other topics studied by researchers in this area include how organizational culture affects innovation management (Huarng et al., 2021), the impact of strategic knowledge management on innovation (Marques et al., 2016; Nakshabandi and Jasimuddin, 2018), the social processes that take place during the implementation of radical organizational innovation (Keränen et al., 2022), and the determination of the necessary organizational methods for generating innovation within an enterprise (Vargas-Halabi et al., 2017). According to Aziz (2022), in today's fast-paced society, people are finding less and less time and space to create changes that will stay. As a result, governments, communities, regulators, and lawmakers should develop new guidelines to aid individuals and businesses in implementing sustainable marketing innovation (SMI). They should invest in consumer education and assistance programs to help consumers change their social values and intervene with companies to help influence consumer choices in a way that benefits society.

As demonstrated in Figure 1 by Naeini et al. (2022), the timeframe (2004–2020) has eight clusters. There are two clusters in the first quarter (One bordering the fourth quarter) and two in the second, third, and fourth quarters. Several substantial adjustments have been made in the clusters and regions associated with each in this time compared to the previous two periods. Important developments in this era include a more profound integration of the keywords and domains of each cluster, an increase in the repetitions of words in each group, and the development of new fields.

### 2.1. Innovation's role in driving economic development

As mentioned earlier, much has been written about the correlation between innovation and economic development. Four different theoretical frameworks can describe this connection. The first school favors a supply-leading notion that innovation Granger generates economic expansion. Supporters of this school of thought maintain that the creation of novel ideas, such as those developed through R&D and other forms of innovation, can lead to the introduction of new goods and services as well as the development of novel processes as well as novel forms of doing business (see, for instance, Pradhan et al., 2018). The second view holds that increased prosperity is a direct source of new technological advancements, known as the “demand-following” theory. It is argued that prosperous nations put more resources into R&D to preserve their economic competitiveness in the global market as they grow (see, for instance, Pradhan et al., 2016). Third, the feedback hypothesis proposes that technological progress and economic expansion mutually cause and reinforce one another (see, for example, Galindo and Méndez, 2014; Pradhan et al., 2016; Wang and Fu, 2023). Intriguingly, depending on the factors and samples employed, researchers such as Pradhan et al. (2017) found evidence for all three theories. The fourth school of thinking presents a neutrality hypothesis, contending that technological progress and economic expansion are not Granger-caused (Pradhan et al., 2017) because many of these economies might be in their formative stages of invention. As a result, innovation has little or no impact on economic development. Traditional factors of production undoubtedly account for much of the growth in these economies.

### 2.2. Entrepreneurship's role in driving economic development

Four schools of thought explain the Granger causality between entrepreneurship and economic growth, which is analogous to the relationship between innovation and economic expansion/development. A supply-leading theory first suggests that entrepreneurial Granger generates economic expansion. The economic justification is that business owners take risks and invest in the R&D that leads to the introduction of brand-new goods and services, as well as enhanced versions of existing ones and innovative new ways of doing business (Urbano and Aparicio, 2016; Sedera et al., 2022). Secondly, many believe entrepreneurs directly result from economic progress (the “Granger cause” view). This line of thinking is premised on the idea that as an economy develops, governments can better invest in fostering entrepreneurship by putting in place fiscal and non-fiscal incentives, new institutions, and a more favorable regulatory framework for business creation. Boosting entrepreneurial efforts and innovative ideas through strengthening business support infrastructure. Nguyen and Nguyen (2023) and Pan et al. (2023) conducted two studies that provide credence to this theory. There is also the feedback hypothesis, which states that entrepreneurial activity and economic expansion mutually cause and reinforce one another via the process of Granger causation. For instance, fostering an entrepreneurial mindset may boost the economy, and a flourishing economy can inspire more risk-taking and new business creation. Huggins and Thomson (2015) and Wennekers and Thurik (2016) are two pieces of research that lend credence to this theory.

### 2.3. Interactions between innovation and entrepreneurship

Thirdly, a body of research looks at the probable Granger causation between entrepreneurship and innovation, and these findings may be summed up in four distinct ways. The supply-leading hypothesis advocates claim that entrepreneurs are the Granger cause of new products and processes. Economic theory suggests that business owners who risk capital on R&D and innovation are more likely to get a positive return on their money. These business owners have a knack for determining which companies will provide them with the best return on investment. Entrepreneurs who advocate for changes drive most of the venture capital business in many industrialized economies. According to the proponents of the demand-following theory, entrepreneurship is caused by innovation via the Granger causality chain. They justify this by saying that new advances, particularly technical ones, have led to new forms of company organization. These advancements have produced new “open innovation” platforms and made it easier to enter new markets (Jasimuddin and Nakshabandi, 2019). The latter has further strengthened the increased availability of information, expertise, market intelligence, and other resources, all of which have contributed to a rise in entrepreneurial endeavors. Investments like Uber's online user-friendly platform connecting taxi drivers have allowed every driver to try their hand at entrepreneurship. Small Business Innovation Research (SBIR) and other government initiatives in the United States are shown to have positive knock-on effects (Zhang et al., 2023). The SBIR program has facilitated the rise of new startup companies and commercialization endeavors by giving small enterprises access to research, development, and innovation funding.

Another study lends credence to the idea of an entrepreneurship-innovation nexus feedback theory, which holds that the dissemination of entrepreneurship and innovations are Granger-caused by one another. The idea here is that when business owners spend money on modern technologies, such advancements improve the availability and quality of previously available goods and services while lowering entry barriers. These factors encourage new business owners to enter the field. Galindo and Méndez (2014) and Capello and Lenzi (2016), for example, examined the connection between entrepreneurship and innovation (2013). Fourth, the neutrality hypothesis states that entrepreneurialism and innovation do not Granger-cause one another. One economic justification is that some nations have poor or non-existent entrepreneurial and innovation ecosystems. Over-regulation, hostile business practices, rent-seeking conduct, poor expenditures in R&D, and a lengthy procedure to register patents and company licenses/permits may all impede innovation and entrepreneurial activity. These factors are apparent in nations like these (Pattinson et al., 2023).

## 3. Papers in the Research Topic

Following that, we hereunder present a review of each article accepted for this Research Topic. After that, we establish an agenda for innovation management and entrepreneurship development regarding the current opportunities and challenges, interspersing our opinions with those of our contributing author teams. Our goal is to inform Frontiers of Psychology's readers about the theories, methods, and best practices that connect entrepreneurship, innovation, and economic growth. We are pleased to receive 12 submissions for the peer-reviewed papers. We thank those who volunteered to review the submitted manuscripts and met a tight deadline.

***The first article*** in this Research Topic explores how variations in leadership style affect workers' propensity for unconventional thinking-considering both internal and external subordinate viewpoints (Lu et al.). Based on the differential sequence pattern, the authors argue that differential leadership emerged as a decentralized form of leadership through time. Both “insider subordinates” and “outer subordinates” can benefit from the dynamic changing of roles. The study uses game logic to distinguish between the strategies employed by an insider and outer subordinates while enacting deviant, creative actions. Through a reasoning process aided by a simulation graph, individuals with a high risk-taking trait on the inside and employees with a high internal control personality on the outside are both encouraged to engage in deviant creative activities. Differential leadership fosters workers' deviant, innovative conduct, and the theoretical derivation of behavior gives necessary references and countermeasures to encourage this behavior.

***The second article*** in this Research Topic examines how students' perceptions of their abilities affect the degree to which they can innovate and adapt to new social situations in the workforce (Li et al.). The authors argue that a greater degree of creative capability and social adaptation among students increases their employability by giving them greater faith in their talents to take calculated risks and realize desired outcomes. According to the study, positive interference of innovative capability, social adaptability, and self-efficacy may increase undergraduates' employability in the knowledge-based economy era.

The creative work behavior in high-tech enterprises: chain intermediate impact of psychological safety and information sharing is the topic of ***the third article*** in this Research Topic (Xu and Suntrayuth). This study's overarching goal is to examine how psychological safety (PS) and knowledge sharing (KS) might mediate the connection between an organization's innovation environment (OIC) and employees' creative work behavior (IWB). This research, grounded in Social Cognitive Theory (SCT), offers a theoretical framework for investigating creativity at work. The structure of the expanded SCT model was validated using data from 446 R&D professionals at Chinese high-tech companies. There was a favorable relationship between employees' sense of psychological safety and their willingness to take risks at work. Innovation at work was found to be strongly and positively connected with knowledge exchange.

Moreover, psychological safety improves individual innovative work behavior by influencing knowledge sharing among research team members. Psychological safety and knowledge sharing mediate the relationship between organizational innovation climate and work behavior. The study's shortcomings, practical consequences, and suggestions for further research were all examined at length.

The Research Topic's ***fourth article*** looked at Australian organizations to analyze the roles of entrepreneurship and intrapreneurship in converting innovation intention into performance (Abeysekera). The Theory of Planned Behavior provided the theoretical foundation for the study. Entrepreneurship and intrapreneurship were used in the study as mediator constructs to address potential research issues. Data that contrasted performance increases between the COVID-19 financial years 2019–2020 and 2020–2021 were descriptively assessed in the study. Businesses that actively pursue innovation outperform those that do not. With increased performance with business size, major corporations outperformed medium-sized and small businesses. There was no clear distinction between firms with the same or lower performance and those without an innovation-active status. According to the survey, companies have widened their performance approach after the financial crisis to include the triple bottom line, which promotes performance in the economy, society, and the environment.

## 4. Concluding remarks

This Research Topic generates many conversations to advance our understanding of the connectivity of connected entrepreneurship, innovation, and economic growth. The papers in this Research Topic reflect the latest research and empirical findings on all aspects, issues, policies, and practices of innovation and entrepreneurship development in the digital economy of the 21st century at the individual, organizational, industry, national, and international levels.

## Author contributions

All authors listed have made a substantial, direct, and intellectual contribution to the work and approved it for publication.
